# Supplementary figures and images for: Co-Harboring of Beta-Lactamases and mcr-1 Genes in Escherichia coli and Klebsiella pneumoniae from Healthy Carriers and Backyard Animals in Rural Communities in Ecuador
Source: Antibiotics (Basel). 2023 May 5;12(5):856. doi: 10.3390/antibiotics12050856 (PMC10215259; doi:10.3390/antibiotics12050856)

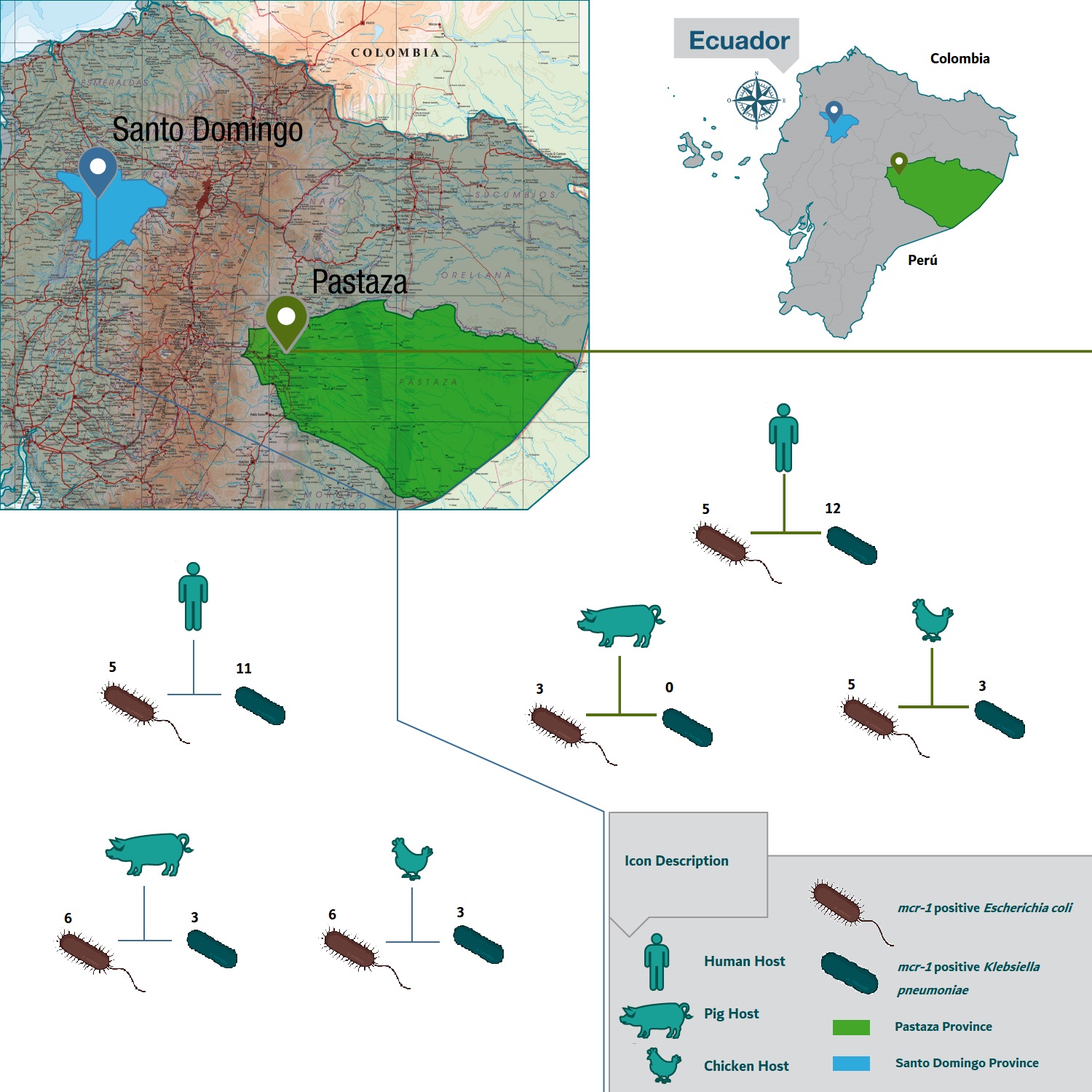

Supplement: Supplementary file 1 [file antibiotics-12-00856-s001.zip › Supplementary Figure S1.jpg]
